# Adherence, Compliance, and Health Risk Factor Changes following Short-Term Physical Activity Interventions

**DOI:** 10.1155/2015/929782

**Published:** 2015-08-25

**Authors:** Lynda H. Norton, Kevin I. Norton, Nicole R. Lewis

**Affiliations:** ^1^School of Medicine, Nursing and Health Sciences, Flinders University, Bedford Park, SA 5042, Australia; ^2^School of Health Sciences, University of South Australia, Adelaide, SA 5000, Australia

## Abstract

*Background.* Low physical activity (PA) levels are associated with poor health risk factor profiles. Intervention strategies to increase PA and quantify the rate and magnitude of change in risk factors are important. *Methods.* Interventions were conducted over 40 days to increase PA in 736 insufficiently active (<150 min/wk PA) participants using either a pedometer or instructor-led group protocol. There were a further 135 active participants as controls. Major cardiovascular and metabolic risk factors, including fitness parameters, were measured before and after intervention. *Results.* Adherence to the interventions was higher for the group versus pedometer participants (87.1% versus 79.8%) and compliance rates for achieving sufficient levels of PA (≥150 min/wk) were also higher for the group participants (95.8% versus 77.6%). Total weekly PA patterns increased by 300 and 435 minutes, for the pedometer and group participants, respectively. Improvements were found for waist girth, total cholesterol, aerobic fitness, and flexibility relative to controls. The change in vigorous PA, but not moderate PA, was a significant predictor of the change in eight of 11 risk factor variables measured. *Conclusions.* Rapid and dramatic increases in PA among previously insufficiently active adults can result in important health benefits.

## 1. Introduction

Humans throughout most of the world are living longer than ever before [1]. It is ironic, however, that as life expectancy continues to increase, chronic illnesses such as diabetes, cardiovascular conditions, and dementia are rapidly rising [2]. This is reflected in increased years lived with disabilities [3]. A number of clearly established and relatively easily measured risk factors are associated with the development of these chronic conditions [4]. Therefore, interventions and behaviours that can impact these health risk factors at both the individual and population levels are targeted and monitored in health promotion strategies [5].

A cluster of major risk factors for adverse health outcomes includes low levels of physical activity (PA), increasing levels of overweight and obesity, and poor metabolic and functional capacity [4, 6]. The importance of these risk factors for health is reinforced by their persistent high ranking among variables contributing to the burden of disease [4, 7].

Public health initiatives typically focus on those at greatest risk of developing, or those with established, risk factors because this is where most gains can often be made.

Low PA has a major influence on a range of other risk factors including body composition, metabolic and cardiovascular health, and fitness and functional capacity. While interventions to increase PA levels among low-active people often result in only short-term adherence [8], there is still an enormous effort to encourage PA to improve health. This is because the prevalence of insufficient levels of PA in many adult populations is above 50% [9–11] and about one-third of the global population is not reaching minimum PA recommendations [12]. Long-term observational studies [13, 14] and numerous systematic reviews and meta-analyses of controlled trials have shown significant risk factor reductions following various types of PA interventions in a general dose-response pattern [15, 16]. What are less well known are the interactions of exercise volume and type, including exercise intensity levels, on adherence and compliance, and health-related physiological responses to new PA programs [17, 18]. For example, it is widely acknowledged that vigorous or high intensity exercise [19] is associated with a greater risk reduction than an equivalent time or total energy expenditure made up of moderate intensity activity [17, 20, 21]. However, further intervention studies are required to quantify the relative contributions of exercise volume, intensity, and type on risk factor changes.

*Aims.* The aims involved insufficiently active adults undertaking short-term intensive PA interventions to determine the (1) adherence and compliance rates, (2) magnitude of risk factor changes, and (3) interactive effects of exercise volume and intensity on risk factors.

## 2. Materials and Methods

This paper reports on the combined results of multiple 40-day PA interventions for insufficiently active adults. The interventions were conducted during the period 2005–2011. There were two stages of this research project: (1) a randomized control study evaluating the outcomes from pedometer and group-based PA interventions, and (2) a nonrandomized study using pedometer or group-based PA intervention strategies. All participants in the two types of interventions have been combined in this paper. The combination of interventions allowed evaluation of adherence and compliance rates for both randomized and self-selected participants. It also allowed quantification of several health risk factor changes following the interventions and their relationships with exercise volume and intensity. The methods and designs of the group and pedometer PA interventions have been described in detail previously and were kept consistent across cohorts [22]. A brief overview is provided here.

### 2.1. Subject Recruitment and Allocation

All participants were recruited from a university, tertiary hospital, and several government departments within a metropolitan region. The institution's research ethics committee approved the study and all subjects gave informed written consent. A total of 871 subjects aged 18–63 years volunteered and undertook preparticipation PA screening. In order to participate in any of the PA interventions subjects had to meet the following selection criteria:

1. be insufficiently active (<150 minutes of weighted PA in the previous week) according to the Active Australia Survey (AAS) [20],
2. satisfy the preexercise screening guidelines, using the Sports Medicine Australia screening system [23].

Further, for the RCT intervention study, the participants had to be willing to either (a) wear a pedometer daily for the duration of the 40-day intervention or (b) participate in the 40-day group-based intervention. Those who achieved ≥150 min/wk and indicated they had been regularly active over the previous 12 months were invited to participate as active controls. All subjects then undertook a formal laboratory orientation to the testing protocols and a second laboratory visit was scheduled for preexercise screening and health and fitness assessments.

### 2.2. Preexercise Screening and Testing

A series of health-related questions and physiological tests were used to identify subjects requiring medical clearance before beginning the 40-day PA intervention. Broadly, people with either signs or symptoms of, or established, disease were advised to seek medical clearance before beginning PA. Additionally, subjects required medical clearance if they had extreme, or multiple, cardiovascular, metabolic, or respiratory system risk factors [23].

Health and fitness-related variables were measured before and after intervention. These included PA patterns over the previous week using the AAS recall questionnaire [20]; anthropometry measures of height, weight, waist and hip girths, triceps, biceps, and subscapular skinfold thicknesses [24], resting blood pressure (Dinamap Pro 100), grip strength (Takeikki, Japan), sit and reach flexibility, and fasting total cholesterol using finger-tip blood samples and a Reflotron Plus analyzer (Hoffman La Roche Ltd., Basel, Switzerland) were also collected. A submaximal cycle ergometer test was undertaken that involved 3 × 3 minutes stages to approximately 75% predicted HRmax [25]. This was used to estimate maximal aerobic fitness (VO_2max⁡_). Reliability testing was performed for all variables using ten repeat trials on the same person over consecutive days. Coefficients of variation results were as follows: anthropometry measures: <3.5%; SBP: 4.8%; DBP: 6.8%; strength: 1.7%; flexibility: 8.8%; total blood cholesterol: 7.9%; and aerobic fitness: 2.6%. The reliability for the AAS has previously been shown to have greater than 90% agreement in repeat trials [26].

### 2.3. Physical Activity Interventions

Briefly, the two types of PA intervention were (1) a pedometer-based strategy, wherein participants were instructed to achieve at least 5,000 steps/every day in week one and increase this by 1,000 steps/wk to 10,000 steps/day by week six; and (2) a group-based strategy requiring participants to attend instructor-led activities three times/week (Monday, Wednesday, and Friday) and undertake individual activities for at least 30 minutes on all other days of the week. To verify whether self-report measures of PA changes reflected objectively determined changes in PA, the relationships between PA time reported using the AAS and either the PA measured using HR monitor recordings (*n* = 142) or using pedometer step counts (*n* = 188) were determined.

Both arms were conducted over 40 days following the preintervention testing. Subjects were issued PA diaries and either a pedometer, for the pedometer-based strategy, or a heart rate (HR) monitor (Polar 610s) for the group-based strategy participants, respectively. They were asked to record their activity patterns including activity time and either step count each day or average heart rate for all sessions undertaken. Heart rate monitors were also downloaded weekly for automated analysis of exercise patterns. These records were used to assess compliance with daily exercise prescription. Compliance was measured in two ways: (1) the proportion of participants who achieved the prescribed level of activity each day of the 40-day program (using the diary step counts for the pedometer participants or HR monitor downloads for the group-based participants) and (2) the proportion of participants reaching sufficient levels of weekly PA by the end of the interventions according to national guidelines (≥150 min/wk) using the AAS [20]. Adherence was defined as the proportion of participants who returned for postintervention testing.

**Figure 1:**
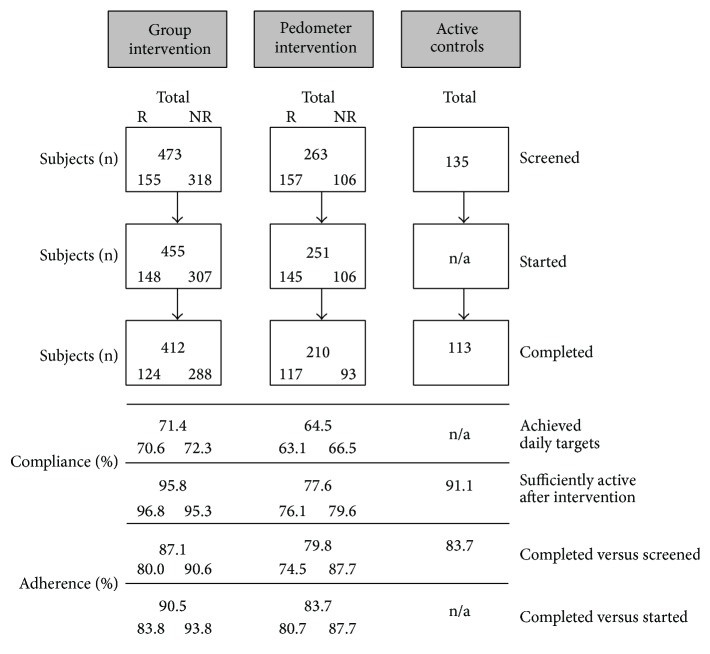
Flow of participants with compliance and adherence percentages for the different study arms and for randomised (R) versus nonrandomised (NR) participants. Compliance was calculated as the proportion of (1) total intervention days the participants achieved the prescribed daily activity targets and (2) participants sufficiently active at postintervention. Adherence was calculated as the proportion of participants remaining in the program at each stage shown.

Given the fact that the volunteers had a low activity base, both interventions started conservatively and progressed in intensity and/or volume over the duration of the programs. No effort was made to control for total energy expenditure or maximum minutes of exercise undertaken. The active controls undertook testing only and were given no instructions about their PA patterns in between testing times. The flow of participants into the three research arms is illustrated in Figure 1. It also shows the total number of participants in each of the PA intervention arms and the breakdown of random versus nonrandom subjects. Randomisation for the RCT was conducted after health and fitness testing. Subjects were assigned to either the group-based (*n* = 155) or pedometer (*n* = 157) intervention arm using computer-generated numbers. There were 135 active control subjects who undertook the pre- and postintervention testing. All other participants (*n* = 424) chose which intervention arm they would join.

### 2.4. Postintervention Testing

Postintervention testing was identical to preintervention testing and participants were scheduled to attend the laboratory within seven days of the program conclusion.

### 2.5. Statistical Analysis

Statistical analysis was performed using Statview software (Abacus Concepts Inc., CA). Differences in compliance and adherence proportions were assessed using either Chi square for raw data or *z*-tests for population proportions. Analysis of covariance (ANCOVA) was used for within- and between-subject comparisons for changes in a range of health risk factor variables. Age was used as a covariate due to a small but significant younger age for the group-based participants. Because this study addresses risk factor changes, analyses were performed on a per-protocol basis where only those participants who completed the intervention were included. Stepwise multiple regression was used to determine the relationships between predictor variables including PA patterns (number of vigorous or moderate sessions, vigorous min PA, and moderate min PA), age, and initial risk factor values and outcome variables when randomly assigned participants from both intervention arms were combined. In some cases other plausible independent variables were also included as described. Multiple regression was also used to determine the ratio of vigorous: moderate PA beta-coefficients for predicting changes in outcome variables. For these analyses the preintervention measure for each variable and age were also included as covariates. Significance was set at a probability level of 5%.

## 3. Results

Figure 1 shows compliance and adherence rates across the intervention period for the three study arms. Compliance with daily PA targets was higher for the group versus pedometer participants (*z* = 1.8; *P* = 0.039). Among participants completing the group intervention program, compliance to daily activity was higher for instructor-led sessions compared to prescribed individual sessions (Chi square = 365; *P* < 0.001). At postintervention testing 95.8% of the group versus 77.6% of the pedometer participants reported being SA (*P* < 0.001; 86.8% and 64.9%, resp., using starters and intention to treat). There was no difference in compliance for sufficient activity levels for nonrandomised versus randomised participants within intervention arms.

Adherence rates were higher among the group compared to pedometer participants (completed versus screened *z* = 2.4; *P* = 0.017 and completed versus started *z* = 2.5; *P* = 0.013). They were also higher for nonrandomised versus randomised participants within the group intervention arm (*z* = 3.2; *P* < 0.001), but not for the pedometer participants (*z* = 1.4; *P* = 0.086).

Table 1 shows the pre- and postintervention descriptive data for the intervention and control participants, respectively. There were dramatic increases in the levels of PA reported by both intervention groups following the 40-day programs. There was a moderate correlation between self-report and objective measures of PA using the HR monitors (*r* = 0.44; *P* < 0.001) and for the pedometer step counts (*r* = 0.36; *P* < 0.001).

**Figure 2:**
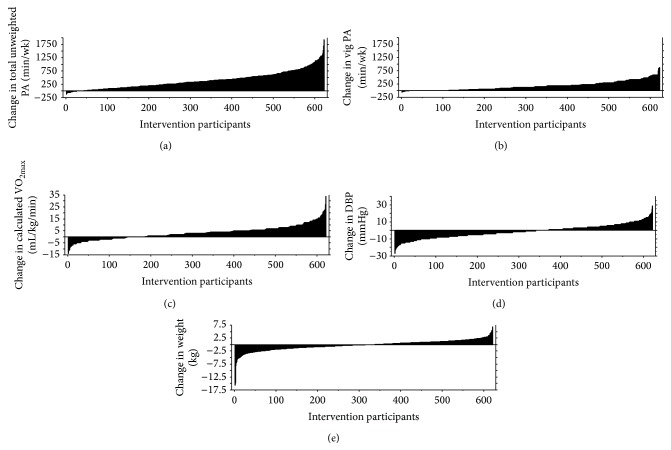
Histograms showing the changes in total PA, vigorous PA, VO_2max⁡_, DBP, and weight across the 40-day interventions. Participants in both treatment groups have been included.

Table 1 shows that the PA increases were significantly greater for the group-based participants. The control participants' PA levels were extremely stable across the study, although there was a large range among individuals within both the intervention and control groups. Pre- and postintervention changes varied among participants within both intervention arms.  Figure 2 illustrates the range of change for several variables. While the vast majority increased their PA patterns (Figures 2(a) and 2(b)) there were not always corresponding individual changes in other health risk factors, for example, VO_2max⁡_ (Figure 2(c)), DBP (Figure 2(d)), and weight (Figure 2(e)).

Repeated measures ANCOVA showed significant intervention group × time interactions for both total and vigorous PA, waist girth, sum of skinfolds, cholesterol, aerobic fitness, grip strength, and flexibility. Table 1 shows all improvements were greater in the group-based participants with the exception of flexibility improvements where there was no difference between the intervention arms.

Multiple regression analyses involved intervention participants who were randomly allocated. Stepwise regression indicated the change in every dependent variable was a function of the preintervention value although the variance explained was generally low-moderate (Table 2). Overall, participants having a poorer starting level were more likely to improve across the intervention. Age was also an independent predictor in several risk factor changes. Furthermore, the changes in weight, BMI, waist and hip girths, skinfolds, cholesterol, aerobic fitness, and flexibility across the interventions were significantly related to the change in minutes of vigorous PA per week but not the level of moderate PA. Walking and total moderate PA minutes and number of sessions of moderate or vigorous PA did not appear in any regression models indicating that they did not contribute significantly to the prediction of risk factor changes. A comparison between these results and when all intervention participants were combined (*n* = 622) showed remarkably similar patterns. The larger group showed that every equation had the same predictor variables with the exception of four of the five anthropometry models where age became a significant predictor and in the flexibility model where vigorous PA did not contribute significantly.

Multiple regression analyses to predict risk factor changes, and which forced inclusion of both vigorous and moderate PA, showed the ratios of beta-coefficients were large and always in favour of vigorous activity. These ratios were 2.0, 3.5, 4.6, 5.3, 6.0, and 11.9 for waist, aerobic fitness, hip, cholesterol, skinfolds, and weight, respectively.

## 4. Discussion

This study involved 622 insufficiently active adults completing 40 days of daily PA in either a pedometer or instructor-led intervention. A further 113 habitually active participants acted as controls. Total weekly PA patterns increased by 300 and 435 minutes, for the pedometer and group participants, respectively. Several common health risk factors were measured before and after intervention. Most risk factor variables changed in the direction of improved health. In general, the poorer the starting levels, the greater the extent of change. Of most importance was the finding that the change in minutes of vigorous PA per week was a significant predictor of the change in eight of 11 risk factor variables. On the other hand, the level of moderate activity was not an independent predictor in any of the risk factor changes measured. Similarly, the number of sessions per week of moderate or vigorous PA did not predict changes in risk factor variables beyond that of vigorous PA volume changes.

### 4.1. Compliance and Adherence

The levels of both compliance and adherence in the intervention arms of the present study were high relative to many other interventions. In general, however, short-term interventions such as the present study tend to show higher rates of adherence and compliance and these drop off as intervention duration increases [27, 28].

Notwithstanding, the current interventions differed from many other intervention types in that participants were required to commit to daily PA, in part, to determine the magnitude and rate of health risk factor changes.

There was a higher compliance in achieving daily targets and also sufficient PA levels among group-based versus pedometer participants (refer to [22] for further detail). This pattern is often found and is likely associated with the social connections and group-cohesion that develop and help motivate people within groups compared with individualised exercise programs [29]. This is reinforced by higher compliance rates found among the group participants for instructor-led sessions versus individual sessions. An interesting aspect of the analysis was the higher adherence rates between randomised and nonrandomised participants, in general, and specifically in the group intervention arm. The ability to choose an intervention arm resulted in higher adherence compared to randomisation but, for those remaining in the program, there was no difference in compliance with daily PA prescriptions.

### 4.2. Physical Activity

The dramatic increase in PA for the intervention participants was to act as a rapid stimulus to determine the extent of health risk factor changes among insufficiently active adults. At the completion of the intervention the overall patterns of PA shown in Table 1 were well above the long-term threshold recommended for health benefits (≥150 min/wk), although they mirrored the high PA patterns of the active controls (approximately 500 min/wk unweighted PA).

The reported PA increases in other studies using low-active adults have ranged considerably. Ogilvie and colleagues [30] in a review of 48 interventions report that the most successful programs can increase walking by up to 30–60 minutes per week. Studies focusing on weight loss encourage greater PA increases, for example, from 179 min/wk [28] up to 280 min/wk [31]. It is often difficult to compare changes in PA across studies because of differing reporting methods. Using effect sizes, meta-analyses on PA interventions have shown variations from 0.28 [14] to 0.52 [32]. By comparison, using our total unweighted PA patterns, the effect sizes were 10.6 and 7.0 for the group and pedometer arms, respectively, although clearly over a relatively short time frame.

The significant difference between the intervention arms was in the level of vigorous PA that, on average, was about three times greater in the group participants.

### 4.3. Anthropometry

On average, the energy expenditure across the 40-day program was approximately equivalent to 28,000 kJ and 58,000 kJ, for the pedometer and group-based participants, respectively [22]. Given the fact that there was no focus on dietary change or weight loss, this suggests that a decrease of about 0.75 and 1.6 kg, respectively, might be expected. Table 1 shows that the intervention weight loss was much smaller and there were no differences across groups. However, there were differences in waist and skinfold measures where losses were greater for the group participants. This is possibly due to the inclusion of resistance type activities as part of the group PA program that may have led to some lean tissue gains. Ostensibly, these differences in anthropometric changes between the interventions parallel differences in exercise volume (changes in PA minutes), as others have demonstrated [28, 33]. However, multiple regression analyses in Table 2 showed that all five anthropometric variable changes were predicted by the initial values and the minutes of vigorous activity reported. Neither the total minutes of all activity nor number of exercise sessions contributed to any of these prediction models. The significant higher-intensity versus fat loss relationship found is supported by other studies [18, 34]. These showed a greater fat loss with high-intensity exercise versus moderate exercise when equated for overall energy expenditure. However, this has not been found in all studies [21]. The variation in weight loss is also worth noting (Figure 2). Across the 40-day program, the largest weight loss was 15.75 kg (pedometer participant) while the largest gain was 5.6 kg (group-based participant). Without knowledge of the food intake it is impossible to explain individual patterns although the participant with the largest loss reported deliberately choosing smaller serving sizes and averaged 9,446 steps/day across the entire 40-day program.

### 4.4. Blood Pressure

Clinical trials have demonstrated that relatively small decreases in blood pressure (~2 mmHg) can significantly reduce cardiovascular disease and mortality [35]. The present study showed no intervention-induced change in blood pressure over the 40-day PA intervention. However, meta-analyses have shown that PA reduces blood pressure in normotensive and hypertensive adults. Furthermore, these changes have been shown to be independent of weight loss [36, 37].

### 4.5. Total Cholesterol

Serum cholesterol levels are correlated to long-term risk of coronary heart disease and cardiovascular disease mortality over a broad range of cholesterol values [38]. The present study found rapid and significant change in total cholesterol for the group-based participants and this was related to the increase in vigorous but not moderate levels of PA. A similar pattern of intensity-related change (−0.55 ± 0.81 mmol/L) was shown following a 24-week PA intervention [18]. The cholesterol change in the present study was also related to the degree of weight loss, although these associations are not always found [21, 39].

### 4.6. Aerobic Fitness

Aerobic fitness has been shown to be a powerful and independent risk factor for all-cause and cardiovascular mortality [6, 7, 40, 41]. For example, a number of studies have shown a 10–25% improvement in survival for each 1 MET increase in aerobic capacity [42]. Aerobic fitness increased by an average of 5.1% (0.4 METS) and 16.2% (1.3 METS) in the pedometer and group participants, respectively. The gains in fitness are in line with other interventions for low active adults which have generally improved from approximately 5–22% [18, 33, 43, 44]. However, given the fact that these other interventions ranged from 12–26 weeks our study indicates that substantial changes can occur far more rapidly.

It is almost invariable that higher intensity exercise training leads to greater changes in aerobic capacity [18, 21, 33, 43]. On the other hand, there appears more uncertainty about the relationships between exercise intensity and other health risk factors including blood pressure, lipid profile, and body composition changes [45]. Notwithstanding, in recognition of the generally greater physiological returns, population PA surveys often weight vigorous activity minutes by two when calculating threshold PA levels for health benefits [20, 46]. Table 2 supports this additional benefit and shows that vigorous, but not moderate, minutes were predictive of changes in aerobic capacity. However, the beta-coefficients show that for every minute of vigorous PA there was a 3.5-fold greater VO_2max⁡_ change when compared to moderate intensity activity, far larger than currently assumed. Given the range in ratios for the risk factor variables analysed, an argument could be mounted that the summation of health benefits associated with vigorous PA is considerably more than double that of moderate PA.

### 4.7. Strength

Poor grip strength and declines in strength over time are both associated with increased all-cause mortality [45, 47, 48]. Furthermore, Warburton and colleagues [49] documented the positive associations between musculoskeletal fitness and mobility, metabolic homeostasis, bone health, and psychological well-being. The present study showed increases in strength for the group participants but, as expected, not for the pedometer participants. These changes were only modest (ES = 0.17) and less than what others have reported in short-duration interventions [50]; however there was not a specific focus on resistance training for strength improvement in our interventions.

### 4.8. Flexibility

Sit and reach flexibility is an index of lower back and hamstring range of motion. Preintervention measures showed that insufficiently active subjects performed poorly versus active control subjects. A learning pattern was evident for flexibility among all subjects although there were intervention × time interactions showing greater changes in the intervention participants. In general, the greatest gains were obtained for the intervention participants with lower baseline levels of flexibility and in those who were younger. Overall, the results support the current ACSM recommendations that encourage flexibility exercises at least twice a week to improve range of movement and musculoskeletal fitness and enhance quality of life [51].

### 4.9. Limitations

This paper included both randomised and nonrandomised participants when reporting health risk factor changes in the intervention arms. This means the differences between the intervention arms may have been biased by individual preferences, but this was not the main focus of the paper. The analysis of exercise intensity versus physiological adaptation involved combining all randomly allocated participants, irrespective of their intervention type. Active controls were used as a stable reference group for PA because (1) being physically inactive is a known health risk and (2) numerous studies have shown that wait-listed or inactive controls often make substantial changes to their activity patterns [52, 53]. Even the active controls in the present study showed that some measures are particularly prone to a learning effect. Furthermore, self-report measures of PA change may be subject to social desirability bias; however, our checks against objectively measured PA showed modest but significant correlations.

The physiological changes reported are those found after 40 days of intervention. While some of these changes are substantial and represent important improvements in health status, the following is not known: (1) how maintenance of the increased PA may continue to impact risk factors, (2) how long these improvements need to be sustained in order to benefit from reduced morbidity and mortality, and (3) if and how it is possible to maintain these high levels of PA among previously insufficiently active adults.

## 5. Conclusions

This study has shown that rapid and dramatic increases in PA among previously insufficiently active adults are possible in short-term interventions. It demonstrated significant differences in adherence among participants randomly versus nonrandomly assigned but no differences in compliance for those remaining in the programs. Significant compliance differences were found for instructor-led versus individual sessions. The study also showed that substantial health benefits accompanied these behaviour changes, even in such a short period of time. Individual responses to the increased PA patterns varied. In general, however, the intensity of the PA had the greatest impact on the magnitude of health risk factors.

## Figures and Tables

**Table 1:** Descriptive data for variables measured before and after intervention. Results show mean (median) ± SD for the two intervention arms and control participants.

Variable	Group					Pedometer					Active controls					Intervention × time
	*n*	pre	SD	post	SD	*n*	pre	SD	post	SD	*n*	pre	SD	post	SD	*P*
Age		36.4	12.9				40.5	12.5				40.5	11.8			
Gender (% F)		71.6					74.3					66.4				
PA total weighted (min/wk)	412	68	45	726	450	210	71	47	439	376	113	743	445	744	629	<0.001^1,2,3^
PA vigorous (min/wk)	412	6	14	229	167	210	7	14	74	105	113	244	209	248	272	<0.001^1,2,3^
Weight (kg)	412	77.7	18.5	77.3	18.0	209	75.7	16.7	75.6	16.5	108	71.7	12.9	71.6	13.0	0.420
BMI	412	26.72	5.30	26.55	5.15	209	26.37	5.57	26.29	5.48	108	24.37	3.30	24.29	3.30	0.287
Waist girth (cm)	412	85.7	14.7	84.5	14.1	209	85.8	14.9	85.3	14.5	108	80.2	10.4	79.9	10.5	<0.001^1,3^
Hip girth (cm)	410	105.3	10.2	104.3	10.9	209	105.4	10.9	105.1	10.8	107	101.0	7.3	100.5	7.1	0.073
Sum of skinfolds (mm)	306	51.3	17.1	48.1	16.0	149	47.7	16.7	46.2	16.6	100	41.5	16.9	39.1	15.8	0.029^3^
Systolic BP (mmHg)	412	122	15.1	121	14.1	209	122	13.9	120	13.0	108	122	12.6	119	15.4	0.479
Diastolic BP (mmHg)	412	75	9.4	73	9.0	209	74	9.8	74	9.3	108	75	9.3	72	9.8	0.096
Total cholesterol (mmol/L)	412	4.7	0.9	4.5	0.8	209	4.9	0.9	4.8	0.9	108	4.8	0.9	4.8	1.0	0.002^1,3^
VO_2max_ (mL/kg/min)	412	27.2	6.8	31.6	7.8	209	27.2	6.9	28.6	8.2	108	37.2	11.3	37.8	10.9	<0.001^1,3^
Grip strength (kg)	304	33.3	9.1	34.8	8.8	209	31.7	7.9	32.4	8.0	107	35.3	9.2	36.5	9.5	0.042^3^
Flexibility (cm)	304	4.6	9.4	8.3	8.7	209	2.7	9.0	6.1	8.2	107	6.7	9.7	8.4	9.3	<0.001^1,2^

PA, physical activity; BMI body mass index; BP, blood pressure.

^1^Group change > control; ^2^Pedometer change > control; ^3^Group change > pedometer.

**Table 2:** Stepwise regression to predict health risk factor changes across the 40-day interventions. All randomly assigned intervention participants were combined and the inclusion criterion was a significant contribution to the multiple *R* (*P* < 0.05).

Dependent variable change	Regression model	*R*	RMSR	*n*
Weight (kg)	2.341 − (0.033∗*b*) − (0.001∗*c*)	0.308	2.12	241
BMI	0.867 − (0.036∗*b*) − (0.001∗*c*)	0.327	0.68	241
Waist girth (cm)	3.532 − (0.049∗*b*) − (0.002∗*c*)	0.330	2.34	241
Hip girth (cm)	5.248 − (0.052∗*b*) − (0.002∗*c*)	0.372	1.88	241
Sum of skinfolds (mm)	3.171 − (0.091∗*b*) − (0.007∗*c*)	0.314	5.28	177
Systolic BP (mmHg)	32.987 − 0.284∗*b*	0.383	9.68	241
Diastolic BP (mmHg)	23.509 + (0.106∗*a*) − (0.389∗*b*)	0.429	7.50	241
Total cholesterol (mmol/L)	1.463 + (0.007∗*a*) − (0.373∗*b*) − (0.001∗*c*) + (0.049∗*d*)	0.574	0.50	241
VO_2max_ (mL/kg/min)	8.109 − (0.256∗*b*) + (0.009∗*c*)	0.350	5.57	241
Grip strength (kg)	3.800 − 0.088∗*b*	0.276	2.86	241
Flexibility (cm)	5.394 − (0.049∗*a*) − (0.151∗*b*) + (0.003∗*c*) − (0.278∗*d*)	0.425	3.54	241

*a* = age (yr); *b* = initial value; *c* = vig PA change (min/wk); *d* = weight change (kg).
